# Long non-coding RNA PVT1 predicts poor prognosis and induces radioresistance by regulating DNA repair and cell apoptosis in nasopharyngeal carcinoma

**DOI:** 10.1038/s41419-018-0265-y

**Published:** 2018-02-14

**Authors:** Yi He, Yizhou Jing, Fang Wei, Yanyan Tang, Liting Yang, Jia Luo, Pei Yang, Qianxi Ni, Jinmeng Pang, Qianjin Liao, Fang Xiong, Can Guo, Bo Xiang, Xiaoling Li, Ming Zhou, Yong Li, Wei Xiong, Zhaoyang Zeng, Guiyuan Li

**Affiliations:** 1 Hunan Key Laboratory of Translational Radiation Oncology, Hunan Cancer Hospital and the Affiliated Cancer Hospital of Xiangya School of Medicine, Central South University, Changsha, Hunan China; 2 The Key Laboratory of Carcinogenesis and Cancer Invasion of the Chinese Ministry of Education, Cancer Research Institute, Central South University, Changsha, Hunan China; 3 The Key Laboratory of Carcinogenesis of the Chinese Ministry of Health, Xiangya Hospital, Central South University, Changsha, Hunan China; 4 Hunan Key Laboratory of Nonresolving Inflammation and Cancer, Disease Genome Research Center, The Third Xiangya Hospital, Central South University, Changsha, Hunan China; 5 Department of Cancer Biology, Lerner Research Institute, Cleveland Clinic, Cleveland, OH USA

## Abstract

The long non-coding RNA, plasmacytoma variant translocation 1 (PVT1), is highly expressed in a variety of tumors, and is believed to be a potential oncogene. However, the role and mechanism of action of PVT1 in the carcinogenesis and progression of nasopharyngeal carcinomas (NPCs) remains unclear. In this study, for the first time, we have discovered that PVT1 shows higher expression in NPCs than in normal nasopharyngeal epithelial tissue, and patients with NPCs who show higher expression of PVT1 have worse progression-free and overall survivals. Additionally, we observed that the proliferation of NPC cells decreased, and their rate of apoptosis increased; these results indicated that the knockdown of PVT1 expression in the NPC cells induced radiosensitivity. Further, we have shown that the knockdown of PVT1 expression can induce apoptosis in the NPC cells by influencing the DNA damage repair pathway after radiotherapy. In general, our study shows that PVT1 may be a novel biomarker for prognosis and a new target for the treatment of NPCs. Additionally, targeting PVT1 may be a potential strategy for the clinical management of NPC and for the improvement of the curative effect of radiation in NPCs.

## Introduction

Nasopharyngeal carcinoma (NPC) is a malignant tumor arising from nasopharyngeal epithelial (NPE) cells^1–3^. NPC exhibits strong regional and ethnic differences; it is one of the most common malignant tumors in Southeast Asia and Southern China, and every year the increasing number of newly diagnosed cases and the mortalities have gained a lot of attention^4–8^. Radiotherapy is the principal and most effective treatment for NPCs^9,10^. In recent years, studies have found that concurrent chemoradiotherapy significantly improved the survival times of patients with locally advanced NPC, but radioresistance continued to occur in some patients after they received radiotherapy alone, neoadjuvant chemotherapy combined with radiotherapy, or concurrent chemoradiotherapy; these caused local and distant metastases of the tumor and led to poor prognoses^11–13^. Therefore, studying the potential mechanisms of the occurrence of radioresistance in NPCs can provide more effective strategies for clinical treatment and prolong the survival times of NPC patients.

Currently, many studies have investigated the mechanisms of radioresistance, such as DNA damage^14^, tumor stem cells^15,16^, and autophagy^17,18^. In particular, radioresistance due to DNA damage has been extensively studied. Radiation can induce DNA damage in tumor cells, further leading to apoptosis and necrosis in these cells: this is an important method of clinical treatment for tumors. DNA damage is divided into single- or double-strand breaks in DNA and damages in bases. In tumor cells, the damaged DNA is identified by many proteins; some of these bind with the damaged DNA to mask these damaged areas, which consequently are not recognized by the repair system, and this process eventually leads to cell apoptosis. However, some proteins bind with the damaged DNA to initiate the repair pathway, and the enhancement of the ability to repair DNA damage often causes tumor cell radioresistance. Additionally, several studies have observed that the levels of some proteins that are related to radioresistance, including 14-3-3σ, Maspin, the heat shock protein GRP78, manganese-dependent superoxide dismutase^9^, and Raf kinase inhibitor protein (RKIP)^19^, can predict the sensitivity of NPC cells to radiotherapy. In addition to protein-encoding genes, long non-coding RNAs (lncRNAs) also play an important role in radioresistance. lncRNAs are about 200 nucleotides long and are located in the nucleus or cytoplasm of eukaryotic cells^20–24^. Studies have shown that lncRNAs are involved in DNA damage repair^25–27^ and can regulate the differentiation of stem cells^28^. The abnormal expression and regulation of these lncRNAs is closely related to the occurrence and development of malignant tumors^6,24,29–35^. A few studies have shown that lncRNAs have effects on tumor radioresistance^36–40^. These studies suggest that lncRNA may be new potential molecular targets for radiosensitivity.

Plasmacytoma variant translocation 1 (PVT1) is a lncRNA gene, which was first discovered in mouse plasmacytoma in the mid 1980s, and in recent years, many studies have found that PVT1 is involved in the occurrence and development of various malignant tumors. PVT1 shows very low expression in most normal human tissues, but is highly expressed in many malignant tumors and tumor cell lines. For example, PVT1 is highly expressed in tissues and cell lines of gastric cancer^41–43^, non-small-cell lung cancer^44–46^, cervical cancer^45^, and colorectal cancer^47^, and the increase in its expression levels is significantly related to the degree of infiltration of the malignant tumor, the TNM (tumor–node–metastasis) staging, and the regional lymph node metastasis. The above data indicate that PVT1 may be an important cancer gene, and may be involved in the occurrence and development of tumors through various mechanisms. However, the influence of PVT1 on the occurrence and development of NPCs have not been reported yet, and the role of PVT1 in the process of NPC radioresistance has not yet been studied. In our study, we have found that PVT1 is highly expressed in patients with NPC and is related to poor relapse-free survival (RFS) and overall survivals (OS) of these patients. Further studies found that PVT1 may affect the prognosis by regulating radioresistance of NPC.

## Results

### PVT1 is highly expressed in NPC and is associated with poor prognoses in patients with NPCs

**Fig. 1:**
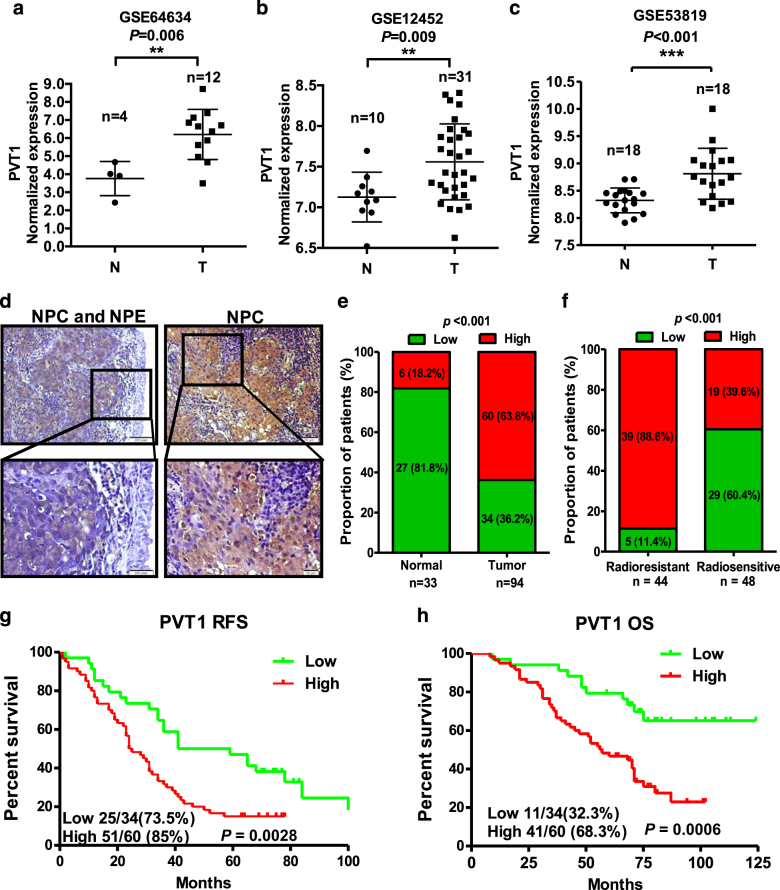
PVT1 is upregulated in NPC tissues and that is associated with poor prognosis. PVT1 was upregulated in the Gene Expression Profiling (GEP) datasets **a** GSE64634, **b** GSE12452, and **c** GSE53819, **P < 0.01, ***P < 0.001. **d** Representative images of the detection of the expression of PVT1 in NPC tissues and adjacent non-tumor NPE tissues by *in situ* hybridization. Upper panel: magnification = ×200, scale bars = 50 μm; lower panel: magnification = ×400, scale bars = 20 μm. **e** The expression of PVT1 in NPC tissues (*n* = 94) is higher than in non-tumor NPE tissues (*n* = 33). **f** The expression levels of PVT1 in the radioresistant and radiosensitive NPC tissues. Treatment data could not be obtained for two patients. Kaplan–Meier survival curves of NPC patients show that NPC patients with high expression of PVT1 (*n* = 60) have shorter **g** RFS and **h** OS than patients with low expression of PVT1 (*n* = 34)

We analyzed normal nasopharyngeal epithelium and NPC tissue samples for differences in lncRNA expression. After obtaining gene expression data for our samples (GSE64634)^22^, we also downloaded another NPC gene expression data from the Affymetrix Human Genome U133 Plus 2.0 platform-based studies in the Gene Expression Omnibus (GEO) database (GSE12452 and GSE53819)^48^. Among the differentially expressed lncRNAs, PVT1 was highly expressed in the NPC samples in all three above-mentioned datasets (Fig. 1a–c). PVT1 expression was detected in paraffin-embedded section samples from patients with NPCs by *in situ* hybridization (Fig. 1d). PVT1 shows significantly higher expression in NPC tissues, compared to the controls with normal nasopharyngeal tissue (64%, 60/94 vs. 18% 6/33; *P* < 0.001; Fig. 1e). The radiosensitivity of patients with high expression of PVT1 was lower than that of patients with low expression (88.6%, 39/44 vs. 39.6% 19/48; *P* < 0.001; Fig. 1f). Patients with NPC who show high expression of PVT1 have shorter RFS (*P* = 0.0028, Fig. 1g) and OS (*P* = 0.0006, Fig. 1h). In conclusion, PVT1 is highly expressed in patients with NPC, and this high expression is associated with poor prognoses in these patients.

### Knockdown of PVT1 enhances the radiosensitivity of NPC cell lines

**Fig. 2:**
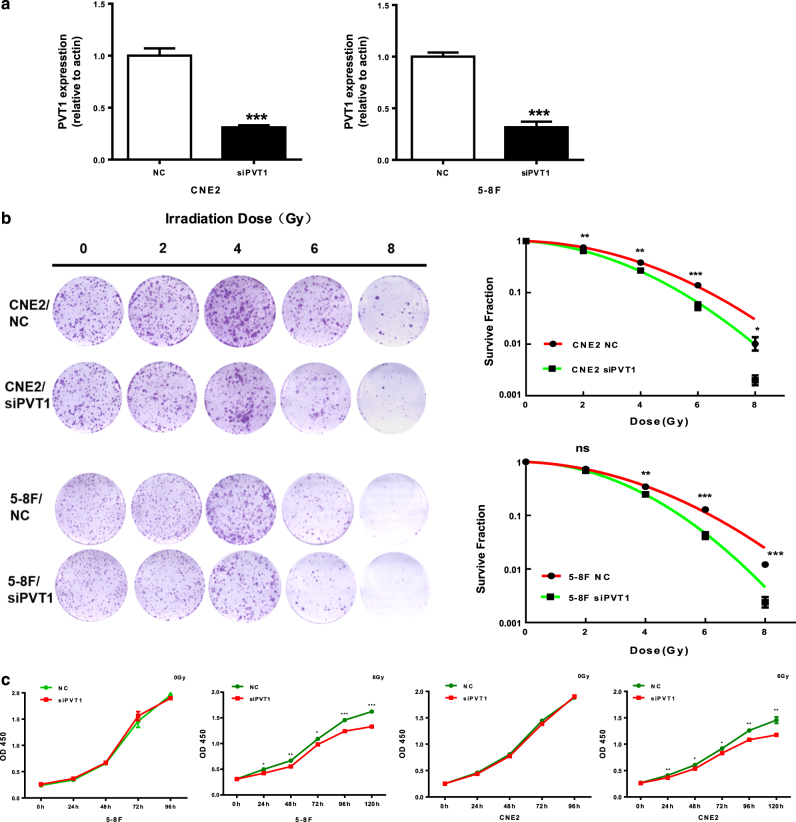
PVT1 knockdown enhances radiosensitivity in NPC cells. **a** Compared to a scrambled RNA sequence (used as negative control (NC)), the siRNA specifically targeting PVT1 (siPVT1) significantly inhibits PVT1 expression in the NPC cell lines CNE2 and 5-8F. **b** Clone formation assay shows that PVT1 knockdown induces radiosensitivity in NPC cells. CNE2 and 5-8F cells were transfected by scrambled RNA (NC) or siRNA targeting PVT1 (siPVT1), and irradiated with 2–8 Gy of radiation; 12 days later the cells were stained (left panel) and dose survival curves were constructed using linear quadratic model analysis (right panel). **c** Proliferation of NPC cells following radiotherapy. In the NPC cell lines 5-8F and CNE2, PVT1 knockdown via siRNA treatment and irradiation with a dose of 6 Gy radiation shows sustained growth inhibition, although PVT1 knockdown alone does not reduce the proliferation of these cells. *P < 0.05; **P < 0.01; ***P < 0.001; ns, no significance.

As radiotherapy is the main treatment for NPC, we reasoned that the clinical outcomes of NPC are closely related to the effects of radiotherapy. Since the high expression of PVT1 is associated with the poor prognoses of patients, we explored whether PVT1 affects the radiosensitivity of NPCs. First, we reduced PVT1 expression by small interfering RNA (siRNA) treatment of the NPC cell lines 5-8F and CNE2. Quantitative real-time polymerase chain reaction (qPCR) results confirmed that the siRNA interfered with and reduced PVT1 expression effectively (Fig. 2a). Next, the cells of the control group (transfected with scrambled RNA) and the PVT1 knockdown group each received irradiation doses of 0, 2, 4, 6, and 8 Gy. At 12 days after exposure, the clone formation test showed that the cell proliferation in the PVT1 interference and control groups showed no significant differences in the absence of radiotherapy; after receiving radiotherapy, the number and sizes of colonies in the PVT1 knockdown group significantly reduced, and with the increase in radiation dose, the clone formation ability further decreased (Fig. 2b). Then, we measured the proliferation ability of NPC cell lines with or without PVT1 knockdown after radiotherapy. The results showed that PVT1 knockdown had no effect on the proliferation abilities of the 5-8F and CNE2 cells that did not receive radiation exposure. However, after exposure to a dose of 6 Gy radiation, the proliferation ability of these cell lines significantly decreased after PVT1 knockdown (Fig. 2c). Finally, these results show that silencing PVT1 in NPC cells can significantly inhibit the clone formation and cell proliferation abilities after radiotherapy and may lead to radiosensitivity.

### Knockdown of PVT1 could promote the apoptosis of NPC cells induced by radiotherapy

**Fig. 3:**
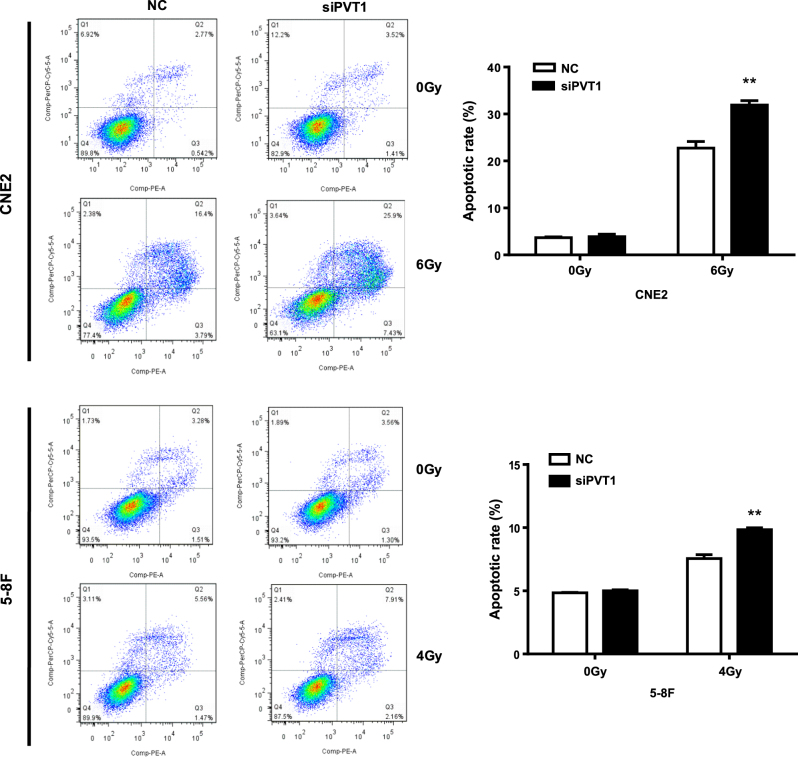
PVT1 knockdown promotes apoptosis in NPC cells after radiotherapy. CNE2 and 5-8F cells were transfected by either scrambled RNA (NC) or siRNA targeting PVT1 (siPVT1), and irradiated with a dose of 6 Gy and 4Gy X-radiation, respectively. Apoptosis in NPC cells was detected by flow cytometry. In NPC cells, PVT1 knockdown promotes apoptosis induced by radiotherapy, although PVT1 knockdown alone did not significantly induce apoptosis. **P < 0.01

The major effect of radiotherapy is the induction of apoptosis in cancer cells. Because PVT1 knockdown enhanced the radiosensitivity and reduced the proliferation of NPC cells after radiotherapy, we investigated whether the radiosensitivity enhanced by PVT1 knockdown occurs via the regulation of apoptosis in NPC cells. We reduced PVT1 expression using siRNAs in 5-8F and CNE2 cells and analyzed cell apoptosis before and after radiotherapy by flow cytometry. As shown in Fig. 3, before radiotherapy, the proportion of apoptotic cells in both the PVT1 knockdown and control groups showed no significant differences; however, after radiotherapy, the proportion of apoptotic cells of the PVT1 knockdown group increased significantly compared to those of the control group. These results showed that interfering with the expression of PVT1 can promote apoptosis induced by radiotherapy in NPC cells, and thus can increase the radiosensitivity in these cells.

### PVT1 knockdown results in diminution of the DNA repair ability through the ATM–p53 (mutated in ataxia telangiectasia–p53) pathway

**Fig. 4:**
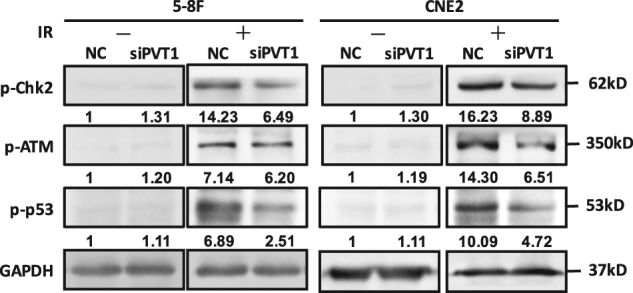
Knockdown of PVT1 can attenuate the ability of DNA repair in NPC cells after radiotherapy. NPC cell lines 5-8F and CNE2 were transfected by either scrambled RNA (NC) or siRNA that targeted PVT1 (siPVT1), and irradiated with a dose of 8 Gy X-radiation. The activation of key proteins, ATM, p53, and Chk2, in the DNA repair pathway was detected by western blot analysis by measuring their phosphorylation levels (p-ATM, p-p53, p-Chk2). Knockdown of PVT1 significantly reduced the phosphorylation levels of these proteins after radiotherapy. GAPDH was used as an internal control.

In tumor cells, the rapid repair of DNA damage after radiotherapy inhibits cell apoptosis, leading to radioresistance. To investigate the effect of PVT1 knockdown on the DNA repair pathway after radiotherapy, we examined the changes in key proteins of the DNA repair pathway in NPC cells after radiotherapy. NPC cells were transfected with either scrambled RNA or siRNAs targeting PVT1; 24 h later, the cells were irradiated at a dose of 8 Gy. After 8 h, the cells were collected to extract protein, and the expression of key proteins on DNA repair pathway, such as phosphorylated ATM kinase (p-ATM), p-p53, and phosphorylated checkpoint kinase 2 (p-Chk2), was detected by western blot analysis. Knockdown of PVT1 significantly decreased the phosphorylation levels of ATM, p53, and Chk2 (Fig. 4). These results indicated that PVT1 knockdown causes a decrease in the DNA repair ability of NPC cells after radiotherapy and enhances their radiosensitivity.

### Knockdown of PVT1 could promote apoptosis through caspases

**Fig. 5:**
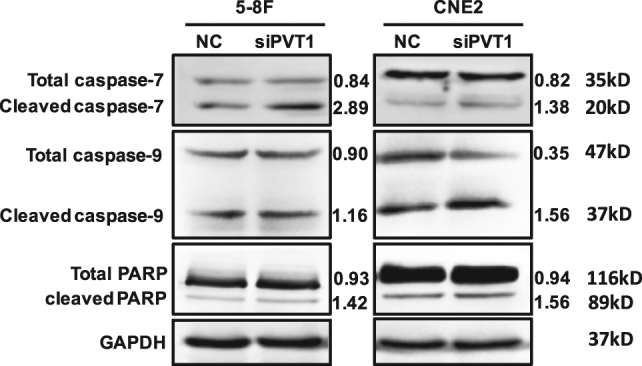
Knockdown of PVT1 activates caspases and PARP after radiotherapy. NPC cell lines 5-8F and CNE2 were transfected by either scrambled RNA (NC) or siRNA that targeted PVT1 (siPVT1), and irradiated with a dose of 6 Gy X-radiation. The activation of caspase-7, caspase-9, and PARP was detected by western blot analysis. PVT1 knockdown significantly increased the cleavage of caspase-7, caspase-9, and PARP. GAPDH was used as an internal control.

Radiotherapy induces DNA damage and triggers apoptosis; caspases, a family of cysteine proteases, are the central regulators of apoptosis. To elucidate the role of PVT1 in cell apoptosis after radiotherapy, we examined the activation of caspase-7, caspase-9, and poly (ADP-ribose) polymerase (PARP), which are the most important regulators of cell apoptosis. The NPC cell lines 5-8F and CNE2 were transfected with either scrambled sequences (as negative control (NC)) or siRNAs that target PVT1 (siPVT1) and exposed to 6 Gy radiation; 48 h later, the cells were collected to detect the expression levels of the cleaved (activated) caspases and PARP^49^. The results showed that the activation levels of caspase-7, caspase-9, and PARP in the cells targeted with siPVT1 were higher than those in the cells targeted by the scrambled sequences. These results suggest that a decrease in PVT1 expression leads to the activation of apoptotic pathway proteins after radiotherapy, thereby enhancing the radiosensitivity (Fig. 5).

## Discussion

The PVT1 gene is located in the human chromosome 8q24 region^50^ near the well-known proto-oncogene MYC. The c-Myc protein encoded by the MYC gene is the hub of many important growth signal transduction pathways^51^. PVT1 has been a hotspot in related studies on lncRNA in recent years. Present studies have found that PVT1 is highly expressed in many tumors. It can inhibit the apoptosis of tumor cells^45,52^, promote cell proliferation by regulating the cell cycle in tumors^44,53,54^, and affect the invasion and metastasis of tumors^55^, which plays an important role in the occurrence and development of malignant tumors. However, its effects on NPCs have never been reported.

In this study, we first investigated the PVT1 expression levels in clinical samples from patients with NPCs. We observed that PVT1 is highly expressed in NPC tissues, and patients with NPC who show high expression of PVT1 have poor prognoses in terms of both RFS and OS. The residual radioresistance in patients with NPC after radiotherapy is the root of NPC recurrence and the leading cause of patient deaths, which implies that PVT1 may affect radioresistance and ultimately lead to poor prognosis of patients with NPC. Therefore, next, we studied whether PVT1 affects the radiosensitivity of NPC. Our results confirmed that PVT1 knockdown can inhibit the proliferation ability and colony-forming ability of NPC cells after radiotherapy, resulting from the increased cell apoptosis.

The biological effects of radiotherapy are mainly DNA strand breakage and cell apoptosis. The common repair pathways of cells include non-homologous end-joining terminal attachment repair based on DNA-dependent protein kinase and the homologous recombination (HR) repair pathway based on the ATM gene^56^. In HR repair, the ATM gene is highly sensitive to radiation^57^. When the DNA double-strand breaks, ATM is activated, damaged DNA complexes are collected to the breaking point, and the DNA repair and apoptosis pathways are mediated through the phosphorylation of downstream products such as Chk2 and p53. Chk2, an important signal transduction protein in the DNA damage pathway, activates a variety of downstream DNA repair genes by phosphorylation. Studies have shown that the increased activation of ATM/Chk2 leads to the enhancement of DNA repair, resulting in radioresistance^58^. Activated ATM can increase the levels of activated p53 protein and the ability for DNA repair. On the contrary, low expression of p-ATM can only partially activate the p53 protein, decrease the repair ability, and lead to cell apoptosis^59^. Our results show that PVT1 knockdown in NPC cells resulted in decreased ATM/Chk2/p53 activation phosphorylation, weakened DNA repair ability, increased tumor apoptosis, and enhanced radiosensitivity.

To investigate how PVT1 influences cell apoptosis during radiotherapy, we examined the activation of some apoptosis-associated proteins. The caspase family are key components in the apoptotic pathway. The activation of caspase factors can trigger the apoptotic pathway, resulting in a cascade effect. Caspase-9, which is in the upstream of the cascade reaction, can be self-activated by the participation of other proteins. Activated caspase-9 can activate caspase-7 downstream; activated caspase-7 combines with a specific substrate to cause cell apoptosis^60,61^. PARP, a key cleavage substrate for caspase-7 binding in apoptotic pathways, can detect the signal of DNA strand breaks, and is regarded as a receptor for DNA damage^62^. Following radiotherapy, the concentration of phosphorylated p53 proteins decreases, and repair functions decline, which can increase the activation of PARP protein cleavage, promoting the apoptosis of tumor cells^63–65^. Our research shows that the knockdown of PVT1 expression following radiotherapy can activate the apoptosis cascade pathway, and significantly enhance the activation levels of caspase-9, caspase-7, and PARP protein. Specifically, caspase-9 activation increases caspase-7 activation, which increases PARP cleavage, leading to an increased rate of apoptosis.

**Fig. 6:**
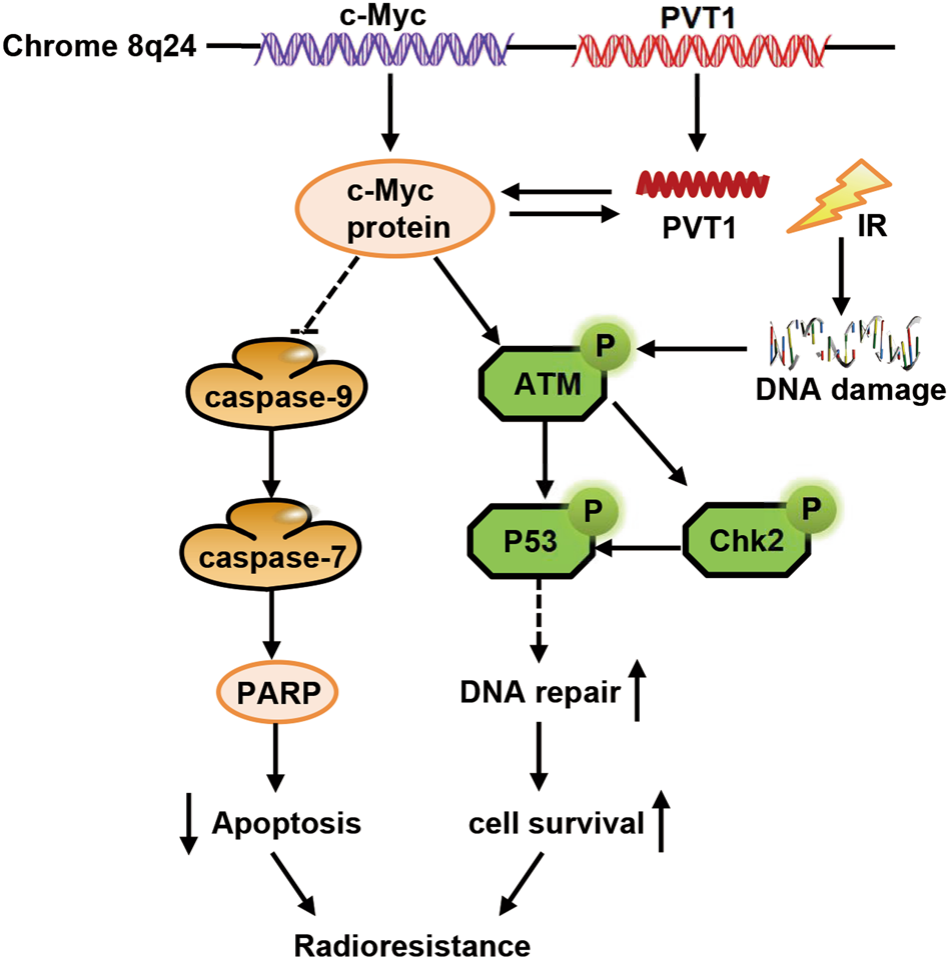
The mechanism of action of PVT1 in the resistance to radiotherapy in nasopharyngeal carcinoma

We have reported, for the first time, the effects of PVT1 on the radiosensitivity of tumor cells, and have preliminarily explored the molecular mechanisms of the influence of PVT1 on NPC radioresistance; however, the specific activation pathways need further elucidation. PVT1 and Myc colocalize in the same chromosome band, and some studies have reported that Myc and PVT1 can synergistically promote tumorigenesis. Myc enhances its own expression by interacting with the PVT1 promoter, and the high expression of Myc may promote PVT1 expression, forming a positive feedback loop. Additionally, PVT1 can prevent protein phosphorylation, which stabilizes the c-Myc protein and promotes tumorigenesis^66,67^. In addition, c-Myc can participate in the repair of DNA double-strand breaks induced by radiotherapy by regulating ATM phosphorylation^68^. Further, PVT1 and c-Myc may interact in NPCs, regulating the activation of ATM phosphorylation that causes radioresistance in NPC. The possible interaction and common mechanisms of action between the DNA repair pathway proteins, PVT1, and Myc, are shown in Fig. 6. Additionally, Myc is closely related to the occurrence and development of NPC, can affect the growth of NPC cells by regulating the expression of microRNA (miRNA)^69,70^, and can participate in the occurrence and development of NPC by affecting the expression of downstream target genes^71^. PVT1 can also regulate downstream target genes by encoding miRNA and can regulate the expression of miRNA by fusing with Myc^72^. PVT1 can also encode a series of miRNAs and regulate the expression of downstream target genes^73,74^. For instance, PVT1 fuses with Myc and enhances PVT1 transcription through the above-mentioned feedback loop, resulting in high expression of PVT1-encoded miR-1204, regulation of downstream target genes of miR-1204, and the promotion of tumor cell proliferation^73^. Further studies can elucidate whether PVT1 regulates miR-1204 in NPC and affects the related genes of radioresistance or directly encodes miRNA and influences the radiosensitivity of NPC.

In normal mammogenesis, PVT1 may function as a competitive endogenous RNA (ceRNA) and interact with the miR-200 family to regulate mRNA expression^75^, but it remains unclear whether it may function as a ceRNA and interact with miRNA to participate in the expression of downstream target genes to regulate NPC radiosensitivity. In addition, PVT1 can regulate the aberrant methylation of p15/p16 gene promoter region by collecting EZH2, downregulating p15/p16^42,76^, and upregulating the ability of promoting cell proliferation and anti-apoptosis of multidrug resistance protein (MDR1), mammalian target of rapamycin (mTOR), multidrug resistance-associated proteins (MRP), and hypoxia/hypoxia-inducible factor-1α (HIF-1α)^77^. In thyroid carcinoma, PVT1 is involved in the formation of thyroid carcinomas by collecting EZH2 and regulating the expression of thyrotropin receptor (TSHR)^78^. Therefore, we hypothesized that in NPC, PVT1 might epigenetically regulate the related genes of radioresistance, thereby affecting the radiosensitivity of NPC.

The role of PVT1 in NPC radioresistance is summarized in Fig. 6. Specifically, PVT1 can promote the radiotherapy resistance of nasopharyngeal carcinoma. First, PVT1 can promote DNA repair by phosphorylation of ATM/Chk2/p53 signaling pathway in NPC cells. PVT1 can also significantly downregulate cleaved caspase-9, cleaved caspase-7, and cleaved PARP, thereby inhibiting apoptosis and ultimately causing radiation resistance.

## Materials and methods

### Data mining and analysis

To identify functional lncRNAs in NPC, we downloaded three sets of gene expression profiling data for nasopharyngeal carcinomas, GSE64634, GSE12452, and GSE53819, from the Affymetrix Human Genome U133 Plus 2.0 platform-based studies in the GEO database. We used the Significant Analysis of Microarray (SAM) software to analyze the differential expression of lncRNAs between the normal nasopharyngeal epithelium and NPC tissue samples in these three datasets^22,32^.

### Tissue samples

Tissue samples of NPC and NPE for this study were obtained from the Xiangya Hospital and the Affiliated Cancer Hospital of Central South University (Changsha, China), including 94 NPC and 33 NPE paraffin-embedded tissue samples for the confirmation of PVT1 expression by *in situ* hybridization. This study was approved by the Research Ethics Board of the Xiangya Hospital and the Affiliated Cancer Hospital of Central South University, and written informed consents were obtained from all patients for research purposes. Clinicopathological data were collected from patient medical records (Supplemental Table 1). All patients with NPC had been histopathologically diagnosed with poorly differentiated squamous cell carcinomas, and had received no previous treatment. Samples of NPE tissues from the patients with chronic inflammation of nasopharyngeal mucosa in the same period were also collected and used for controls.

Patients without distant metastasis received radical radiotherapy alone using a modified linear accelerator in the Affiliated Cancer Hospital of Central South University^79,80^. The total dose of administered radiotherapy was 68 to 72 Gy (2 Gy/fraction, 5 days/week, followed by rest for 2 days). Lymph node-negative patients received a total dose of 60 Gy, and lymph node-positive patients received a total dose of 70 Gy.

The clinical evaluation of radiosensitivity in patients is based on the first re-examination of computer tomography scans after undergoing radiotherapy for a month, according to the following criteria: patients with radioresistant NPC were defined as those with persistent disease (incomplete regression of primary tumor and/or neck lymph nodes) at >3 months, or with local recurrent disease in the nasopharynx or neck lymph nodes or both within 12 months of completion of radiotherapy. Patients with radiosensitive NPC were defined as those who did not experience either local residual lesions over 3 months (complete regression) or local recurrent disease for over 12 months after completion of radiotherapy. The patients were followed-up for 5–10 years. Disease-free survival was defined as the duration from the date of completion of primary radiotherapy to the date when recurrence or distant metastasis was confirmed by clinical evidence or pathological diagnosis. OS was defined as the period that patients were alive from the date of first diagnosis to the date of death due to cancer progression or date of the end of follow-up.

### *In situ***hybridization**

*In situ* hybridization was performed to detect PVT1 expression in NPC specimens using three different nucleotide probes from different regions of PVT1; glyceraldehyde 3-phosphate dehydrogenase (GAPDH) was used as positive control. The sequences of PVT1 probes was as follows: Probe-1: 5′-GGT CGG ACT AGA AAA CCG GTC TTC CTC TAA TTT T-3′; Probe-2: 5′-GAG ACT GTA AAA ACT TCT CAG GTC TTA GGA-3′; Probe-3: 5′-CTC ATA AAA CTC TAA CCT CTT AAT TCT CGG TCA G-3′. The probes were synthesized and labeled with Digoxigenin-11-dUTP (DIG-dUTP) at the 3′ and 5′ ends (Invitrogen, Shanghai, China). *In situ* hybridization was performed as previously described^81^. All sections were independently scored by two pathologists who were blinded to the clinicopathological features and the clinical data.

### *In situ* hybridization evaluation criteria

The intensity and the distribution range of the staining were determined by semi-quantitative treatment^82^. Two pathologists scored the results according to the following criteria. (1) Staining intensity: no observed cell staining was scored as 0; cells with light-brown cell staining as 1; cells stained brown with no background staining, or dark-brown-stained cells with light-brown background staining were recorded as moderately positive, as 2; dark-brown-stained cells with no background staining were recorded as strongly positive as 3. (2) Number of positive cells: no positive cells were scored as 0; less than 25% of positive cells as 1; between 25 and 50% positive cells as 2; positive cells over 50% recorded as strongly positive as 3. The final scores were obtained by multiplying the two scores. The results were as follows: 0 was considered as negative expression, and the final score was 0; 1−2 as weakly positive, and the final score was 1; 3−4 as moderately positive, and the final score was 2; 6−9 as strong positive, and the final score was 3.

All the target cells of each tissue were counted under ×10 magnification, and the counting was repeated twice. The average values obtained from the counting was used for statistical analysis. *In situ* hybridization scores of 2 and 3 indicated high expression of PVT1, and immunohistochemistry scores of 0 and 1 indicated low expression of PVT1. All the samples were scored independently by two experienced pathologists who were double-blinded.

### Cell line and siRNAs

Human NPC cell lines CNE2 and 5-8F were maintained in our laboratory^47,49–53,81^. Cells were grown in RPMI-1640 medium (Life Technologies, Grand Island, NY) supplemented with 10% fetal bovine serum (Life Technologies), and 1% penicillin–streptomycin (Life Technologies) in a humidified incubator with 5% CO_2_ at 37 °C. The sequence of the LINC PVT1 targeting siRNAs was as follows: 5′-CAC UAC UGA CCU UGC AGC UUA UUA U-3′; the sequences of non-target scrambled RNA controls were provided by Invitrogen. For gene knockdown, cells were seeded overnight and transfected either with the siRNAs targeting LINC PVT1 or non-target scrambled control siRNAs (Invitrogen) using Lipofectamine RNAiMAX Reagent (Invitrogen) in OptiMEM medium (Invitrogen).

### Cell proliferation assay

The cells transfected with siRNA were seeded into 96-well plates (500 cells per well), incubated for 24 h, and irradiated with a dose of 6 Gy radiation. According to the manufacturer’s instructions, a Cell Counting Kit-8 (CCK-8) kit (Dojindo Laboratories, Kumamoto, Japan) was used to determine cell proliferation after irradiation. The absorbance of each well was monitored by a spectrophotometer at a wavelength of 450 nm. Each experiment was performed at least three times in triplicate wells.

### Cloning formation assay

A cloning formation assay was used for accessing the radiosensitivity of the NPC cells after incubation. First, the cells were seeded in 6-well plates (500, 1000, 2000, 3000, and 4000 cells per well in triplicate) and incubated overnight. Next, the cells were exposed to irradiation doses of 0, 2, 4, 6, and 8 Gy, respectively. Then, after 12 days of culture, the surviving fractions (>50 cells) were stained with 0.5% crystal violet and counted under a microscope. Finally, the data were fitted to a linear quadratic model to create the cloning formation survival curves to evaluate the radiosensitivity of these cells.

### Cell apoptosis assay

First, the 5-8F and CNE2 cells were seeded in 6-well plates and transfected with siRNA for 24 h. Next, these cells were exposed to irradiation doses of 4 and 6 Gy, respectively. After 48 h, the cells were harvested. According to the protocols, an annexin V-fluorescein isothiocyanate/propidium iodide apoptosis detection kit (BD, San Jose, CA, USA) was used to stain cells and the cell apoptosis was immediately detected by flow cytometry.

### Real-time PCR

Cellular RNA was extracted using TRIzol reagent (Invitrogen, Carlsbad, CA, USA). For performing Real-Time Quantitative Reverse Transcription PCR (qRT-PCR), RNA was reverse transcribed to cDNA by using a PrimeScript RT reagent Kit (Takara, Dalian, China). qRT-PCR was performed using a SYBR_PremixExTaqII kit (Takara, Dalian, China) in the CFX96 Real-Time PCR Detection System (Bio-Rad, Hercules, CA, USA) to determine the relative expression levels of target genes. The sequences of qRT-PCR primers are as follows: LINC PVT1: forward primer 5′-TGG CTG AGA GGG TTG AGA TC-3′ and reverse primer 5′-GCT GTA TGT GCC AAG GTC AC-3′; β-actin: forward primer 5′-TCA CCA ACT GGG ACG ACA TG-3′ and reverse primer 5′-GTC ACC GGA GTC CAT CAC GAT-3′; β-actin was used as reference and normalization control.

### Western blotting

Lysis, electrophoresis, and target protein visualization were performed as described previously. Briefly, 50 μg of cell lysates were separated by 10% sodium dodecyl sulfate–polyacrylamide gel electrophoresis and transferred onto a polyvinylidene fluoride membrane (Millipore, Billerica). To assess protein expression, the blots were incubated overnight at 4 °C with the following primary antibodies: rabbit antibodies against caspase-7, caspase-9, PARP, phospho-ATM (Ser1981), phospho-Chk2 (a serine/threonine kinase) (Thr68), and mouse antibody against phospho-p53 (Ser15) (Cell Signaling, Danvers, MA). After washing with tris-buffered saline-Tween, the blots were incubated with horseradish peroxidase-conjugated secondary antibodies (Cell Signaling Technology) for 1 h at 37 °C. The signal was visualized using enhanced chemiluminescence (EMD-Millipore, Billerica, MA) and quantified by densitometry using ImageJ software (http://rsb.info.nih.gov/ij). GAPDH (Cell Signaling Technology) was used as a loading control.

### Statistical analysis

Data analyses were conducted by the GraphPad Prism 5 (GraphPad, La Jolla, CA). Survival curves were plotted using the Kaplan–Meier method, and compared using the log-rank test. Student’s *t-*tests were used to evaluate significant differences between any two groups of data; one-way analysis of variance was used if there were more than two groups. The dose survival curve was calculated by a linear quadratic model (*Y* = exp(−(a*x + b*(*x*^2)))). All data are represented as means ± standard deviations. *P* < 0.05 was considered statistically significant.

### Consent for publication

The authors confirm that written consents have been obtained from the patients to publish this manuscript.

### Availability of data and material

Clinicopathological data were collected from patient medical records and have been reported in the Supplemental Table S1.

### Conflict of interest

The authors declare that they have no conflict of interest.

## Electronic supplementary material

Supplemental Table S1
